# Raising the ‘Good’ Oxidants for Immune Protection

**DOI:** 10.3389/fimmu.2021.698042

**Published:** 2021-06-04

**Authors:** Alexia Dumas, Ulla G. Knaus

**Affiliations:** Conway Institute, School of Medicine, University College Dublin, Dublin, Ireland

**Keywords:** reactive oxygen species, NADPH oxidase, microbiota, host defense, immune signaling, redox medicine, lactobacilli, glucose oxidase

## Abstract

Redox medicine is a new therapeutic concept targeting reactive oxygen species (ROS) and secondary reaction products for health benefit. The concomitant function of ROS as intracellular second messengers and extracellular mediators governing physiological redox signaling, and as damaging radicals instigating or perpetuating various pathophysiological conditions will require selective strategies for therapeutic intervention. In addition, the reactivity and quantity of the oxidant species generated, its source and cellular location in a defined disease context need to be considered to achieve the desired outcome. In inflammatory diseases associated with oxidative damage and tissue injury, ROS source specific inhibitors may provide more benefit than generalized removal of ROS. Contemporary approaches in immunity will also include the preservation or even elevation of certain oxygen metabolites to restore or improve ROS driven physiological functions including more effective redox signaling and cell-microenvironment communication, and to induce mucosal barrier integrity, eubiosis and repair processes. Increasing oxidants by host-directed immunomodulation or by exogenous supplementation seems especially promising for improving host defense. Here, we summarize examples of beneficial ROS in immune homeostasis, infection, and acute inflammatory disease, and address emerging therapeutic strategies for ROS augmentation to induce and strengthen protective host immunity.

## Introduction

Reactive oxygen species (ROS) is a generic term referring to oxygen-derived compounds capable of reacting with biological molecules through an oxidation-reduction (“redox”) mechanism. ROS include a set of highly reactive radicals (e.g., superoxide anion, hydroxyl radical) as well as non-radical species [e.g., hydrogen peroxide (H_2_O_2_)] produced enzymatically or chemically in eukaryotic and prokaryotic cells. In mammals, one of the main intracellular sources of ROS is the mitochondrial electron transport chain (ETC) during the establishment of the proton-motive force. Premature electron leakage to molecular oxygen occurs mainly from complex I and III of the ETC during the serial transfer of electrons, causing superoxide formation that is converted to H_2_O_2_ by superoxide dismutase (SOD) (1, 2). In contrast to ROS generation as by-product of aerobic metabolism, of oxidation of fatty acids or proteins, or of enzyme-substrate reactions (e.g., xanthine oxidase, lipoxygenase, cyclooxygenase, monoamine oxidase), the NADPH oxidase family (NOX/DUOX) is solely dedicated to ROS production and catalyzes the reduction of molecular oxygen to superoxide or H_2_O_2_. Seven oxidase isoforms have been characterized in humans, differing in their catalytic core (NOX1-5 and DUOX1-2), their requirement for additional components for complex stabilization and/or enzyme activation, their subcellular localization and in tissue specificity (3, 4). The NADPH oxidase prototype is the NOX2 enzyme, an essential superoxide source for pathogen defense by neutrophils and macrophages. The rapid increase of superoxide (‘respiratory burst’) is accompanied by the formation of dismutation and adduct products, including hypochlorite (generated by myeloperoxidase, H_2_O_2_ and chloride ion) and peroxynitrite (spontaneous reaction of superoxide with nitric oxide), both strongly oxidizing and nitrating compounds that drive pathogen killing in concert with activated proteases (5). Tight regulation of ROS generation is essential, as ample and uncontrolled production of highly reactive species over an extended time period will cause irreversible redox modifications on biomolecules, thus promoting oxidative damage.

ROS is not only produced during host defense or in proinflammatory situations. Superoxide and H_2_O_2_ are continuously generated, converted, and degraded in physiological conditions (6, 7). H_2_O_2_ as a key intracellular messenger mediating signal transduction in all cell types maintains homeostasis and physiological host responses (8, 9). H_2_O_2_-initiated redox signaling is essential for basic cellular processes (e.g., proliferation, migration, secretion), thereby supporting complex functions in mucosal immunity (e.g., barrier integrity, host-microbiota communication, host defense, chemokine/cytokine generation, wound repair), driving innate immune functions and regulating adaptive immunity. By aquaporin-facilitated diffusion across the plasma membrane or by release from intracellular organelles (e.g., redoxosomes, microvesicles, mitochondria), H_2_O_2_ mediates directly or more commonly *via* redox relays the reversible oxidation of distinct amino acids, modifying the structure and function of targeted proteins and consequently the activation state of the associated signaling pathways. In mammals, thiol oxidation of certain reactive cysteine residues occurs within redox-sensitive proteins, including phosphatases and tyrosine kinases, leading to activation or inactivation of the targeted protein (2, 10). Redox signaling also drives the antioxidant response to prevent excessive ROS production. A notable conduit for transcriptional activation of antioxidant genes is the KEAP1-NRF2 pathway. H_2_O_2_-mediated oxidation of the redox-sensitive adaptor KEAP1 disrupts its cytoplasmic complex with NRF2, promoting nuclear translocation of newly synthesized NRF2 which will bind to antioxidant response elements (ARE) located in the promoter region of NRF2 target genes such as NAD(P)H quinone oxidoreductase 1 (NQO1), heme oxygenase 1 (HMOX1), glutamate-cysteine ligase (GCL) and glutathione S transferases (GSTs) (11, 12).

Coupled oxidant-antioxidant pathways have been conserved throughout evolution. For example, in bacteria dedicated transcriptional regulators (e.g., OxyR, OhrR, PerR, SoxR) sense ROS/RNS in order to induce an appropriate detoxification response (e.g., radical scavenging and metal sequestrating systems), and to maintain intracellular oxidant levels within a safe limit (13, 14). The H_2_O_2_ sensing transcriptional regulator OxyR is a well-studied example in mainly Gram-negative bacteria. When intracellular H_2_O_2_ exceeds a certain threshold in *Escherichia coli* [~ 0.1-0.2 µM (15, 16)], oxidation of OxyR on two cysteine residues prompts a conformational change due to intramolecular disulfide bond formation, activating the expression of OxyR-dependent antioxidant defense genes (17, 18). At mucosal surfaces physiological H_2_O_2_ levels mediate the intricate redox communication between commensal microorganisms and the host barrier tissue. For instance, bacteria can induce or hinder physiological host signaling, either by releasing H_2_O_2_ in the vicinity of host epithelia (e.g., lactobacilli) or by altering host enzyme-mediated ROS generation *via* secreted compounds or physical interaction with epithelial barrier cells (19–21). Likewise, H_2_O_2_ released by host cells can alter bacterial signaling in response to a pathogenic insult, particularly in low oxygen environments (22, 23).

It is important to distinguish these beneficial redox-mediated mechanisms supporting health from conditions promoting supraphysiological level of ROS and the resulting oxidative damage. To ensure physiological conditions, the activation of NADPH oxidases is usually regulated by multiple inputs (e.g., transcriptional, posttranslational, metal ions, nucleotides) and ROS generation is coupled with decomposing and scavenging systems (e.g., catalase, peroxidases, antioxidants) (2, 4, 21, 24). Ongoing perturbation of ROS levels, either at high or low range, has been associated with various pathophysiological states. Persistent increase of highly reactive species (e.g., superoxide anion, hydroxyl radical, peroxynitrite) has been associated with chronic inflammatory and hyperglycemic (e.g., rheumatoid arthritis, inflammatory bowel diseases, type 2 diabetes), tumorigenic and neurological (e.g., Parkinson’s, Alzheimer’s) diseases. Therapeutically decreasing ROS levels by non-specific enzyme inhibitors or antioxidants has been pursued for many decades (25, 26). However, the ill-defined and generalized action of these molecules limits their efficacy and, in some cases, exacerbated the underlying condition (27–32). More targeted approaches, namely inhibition of a specific ROS source, may provide more benefit. Conversely, permanently decreased ROS generation due to loss-of-function variants in genes encoding for NADPH oxidases or other ROS sources, or in genes providing an essential upstream trigger for a superoxide/H_2_O_2_ generating enzyme, have been linked to various pathologies, including recurrent microbial infections, chronic inflammatory diseases, and autoimmunity (33). In this case, a strategy to restore or enhance ROS may prove beneficial.

Understanding redox regulation of vital physiological processes and how alterations initiate or perpetuate disease is important for developing new therapeutic avenues. In addition to traditional therapeutic strategies aimed at counteracting excessive ROS, contemporary approaches should include preservation or even the elevation of certain oxygen metabolites for physiological purposes. This review will provide selected examples of beneficial ROS in protective host immunity and will discuss emerging therapeutic strategies to preserve or augment ROS with the aim of re-establishing homeostasis and promoting host protection.

## Importance of Oxidants in Protective Immunity

### Homeostatic Redox Signaling

Physiological ROS levels modulate many immune functions by redox-sensitive signaling. Not only H_2_O_2_, but also related second messengers such as nitric oxide and possibly peroxynitrite control signaling responses and thus these short-lived molecules represent an integral part of immune regulation (34–37). All redox-regulated pathways show analogous features, namely inhibitory or activating modifications on proteins and lipids (10, 38, 39), and are conserved across different cell types and organisms. Redox modifications can constrain or stimulate signaling pathways. An example for inhibitory oxidative modifications is dual specificity phosphatase 1 (DUSP1) which will be degraded after oxidation or S-glutathionylation of an active site cysteine, resulting in prolonged MAPK-induced proinflammatory gene transcription (40). Reversible oxidative inactivation of protein tyrosine phosphatase 1B (PTP1B) amplified interleukin (IL)-4 receptor activation (41). On the other hand, redox-dependent oxidation of two cysteine residues in the tyrosine kinase SRC caused structural changes that impacted regulatory tyrosines, enabling SRC kinase activation (42). In airway epithelial cells, epidermal growth factor receptor signaling was linked to activation of the NADPH oxidase DUOX1 and subsequent SRC oxidation (43). Kinase activation can also occur by oxidation-driven dissociation of an inhibitor-protein kinase complex as demonstrated for the complex between thioredoxin and apoptosis signal-regulating kinase 1 (ASK1) (44). The liberated form of ASK1 will then form multimeric complexes with other signaling mediators and positively regulate JNK/p38 MAPK pathways, while itself being further controlled by dephosphorylation and deubiquitination (45). More examples and details on redox regulation of signaling pathways can be found in recent reviews (1, 46).

The initiation of redox signaling occurs mainly after ligand stimulation of receptors (e.g., Toll-like receptors (TLR), G protein-coupled receptors (GPCR), NOD-like receptors (NLR), Fc receptors, chemokine and cytokine receptors, and others) and may include one or several ROS sources concomitantly or consecutively, depending on the context. For instance, redox regulation triggered by TGF-β involves the NADPH oxidase NOX4 as well as mitochondrial ROS (mROS) (47). NADPH oxidases seem to be closely associated with TLR-stimulated pathways where oxidase deficiency either inhibits or enhances host cell responses. Loss of NOX1 in smooth muscle cells increased TLR2-mediated MIP-2 generation but impeded matrix metalloprotease 2 secretion and directed cell migration (48). In epithelial cells NOX4 was reported to associate with TLR4, regulating the transcription factor NF-κB (49). Loss-of-function mutations in any of the five subunits comprising the NOX2 complex cause the inherited immunodeficiency chronic granulomatous disease (CGD) (33, 50). One of the effects of CGD in the immune system is the altered responsiveness to TLR ligands, resulting in augmented cytokine generation and hyperinflammation. Neutrophils, monocytes, and dendritic cells isolated from CGD patients or Nox2 deficient mice generated increased levels of cytokines upon TLR2 or TLR4 stimulation (51–54). A hyperinflammatory phenotype with excessive secretion of pro- and anti-inflammatory cytokines was also observed in CGD B lymphocytes stimulated with TLR7 and TLR9 agonists (55). This cytokine overproduction was associated with hyperactivation of p38 MAPK signaling, which may indicate loss of inhibitory feedback signaling, likely involving phosphatases, when the NOX2 enzyme is inactivated. Increased levels of IFNγ and IL-18 in CGD patient tissues was in part traced to macrophages remaining in a proinflammatory M1 state (56). Innate immune cells derived from CGD patients or mice also showed an exuberant IL-1 response to various inflammasome activating stimuli, while the NRF2-controled antioxidant response was dampened (57–59). The hyperinflammatory CGD phenotype in mice and man is intensified by increased neutrophil recruitment to sites of infection and tissue damage, which was recently linked to feed-forward amplification of neutrophil generated leukotriene B4 upon pulmonary zymosan challenge (60). Hence, deficiency in NOX2 enzyme activity creates a proinflammatory environment that is associated with several chronic inflammatory conditions and can predispose to autoimmune disease (61). The molecular mechanisms underlying various facets of CGD hyperinflammation are still not resolved, but NOX2-derived superoxide seems to be required for the precise regulation of many immune cell functions including gene transcription, autophagy, efferocytosis, and dendritic cell-mediated antigen presentation (61–64).

In recent years, the role of mROS signaling in regulating both innate and adaptive immunity received increased attention (65, 66), although in many cases molecular mechanisms are not well characterized and mROS participation in signaling is mainly inferred by using mitochondria-targeted ROS probes and/or antioxidants. Chandel and coworkers linked early on a TNF receptor-TRAF pathway to an increase in mROS (67), and by now many different stimuli are recognized as triggers of physiological mROS generation (6). Here we summarize recent insights into mROS-mediated immune cell responses. Signaling cascades driven by TLRs stimulate not only NADPH oxidase activity, but also *via* TRAF6 the mitochondrial matrix protein ECSIT that stimulates superoxide production from ETC complex I (68). Others reported recently that pro-inflammatory cytokine secretion by *Listeria monocytogenes*-infected macrophages is contingent on mROS-induced disulfide bond formation in the IKK complex regulator NEMO (69). Activation of the NLRP3 inflammasome by pathogen-associated or danger-associated molecular patterns (PAMPS/DAMPS) is controlled by mROS, resulting in release of the proinflammatory cytokines IL-1β and IL-18 (70, 71). The precise sequence of events that may include a transcriptional priming step, oxidized mitochondrial DNA, and mROS-dependent interaction of NLRP3 with thioredoxin-interacting protein is not yet fully established (72). In macrophages NLRP3 inflammasome activation required xanthine oxidase generated superoxide upstream of mROS generation (73), while studies in superoxide deficient peripheral blood mononuclear cells derived from CGD patients revealed that NOX enzymes are not required for NLRP3-mediated IL-1β secretion (74, 75). Cytosolic retinoic acid-inducible gene I (RIG-I)-like receptors such as RIG-I and MDA5 sense and bind distinct features of viral RNA, then oligomerize, and associate with the adaptor protein MAVS at outer mitochondrial membranes and mitochondria-associated membranes. Oligomerization of MAVS recruits several effector proteins to form the MAVS signalosome, driving IRF3/IRF7-mediated transcription of type I/III interferon and NF-κB-induced proinflammatory cytokine expression. MAVS expression has been linked to NOX2 generated superoxide (76), while others reported functional interactions involving MAVS aggregation, mROS generation, and the cytochrome c oxidase component COX5B that couple the antiviral response to autophagy (77). The NLR family member NLRX1 cannot only interact with MAVS at the outer mitochondrial membrane but is also localized within the matrix and inner membrane of mitochondria where interaction with the ETC complex III associated protein UQCRC2 promotes mROS generation, thereby stimulating transcription factors and JNK MAP kinase (78). The signaling function of the immune response-induced mitochondrial interactome is clearly connected to mROS generation, in physiological and pathophysiological conditions, and many more aspects of mitochondrial immune signaling and connections between different ROS sources influencing each other in these processes can be expected in the future.

### Mucosal Barriers and Oxidants

The mucosal immune system in lung, gastrointestinal tract (GIT), urogenital tract, oral and nasal passages employs structural, chemical and immunological barriers to control the interaction with the adjacent microorganism- and noxious substance-rich environment. The importance of mucosal barriers as host defense mechanism is reflected in these multiple layers of protection together with the constant renewal of barrier epithelia, specialized protective measures such as secretion of mucins or surfactant, and highly efficient repair mechanisms. Superoxide and H_2_O_2_, generated by host enzymes or commensal bacteria, are crucial for various aspects of mucosal barrier maintenance as outlined here in selected examples.

The structural integrity of mucosal epithelial barriers relies on intercellular junctions, in particular tight junctions, and after a breach occurred, on rapid proliferation and migration of epithelial cells for wound repair. ROS regulate cytoskeletal dynamics such as oxidative modification of β-actin and tubulin, RhoA GTPase activation and deactivation depending on the context, redox-induced integrin and focal adhesion kinase (FAK) activation and oxidative modifications on other proteins involved in cytoskeletal rearrangements (79, 80). As many of these physiological processes have not yet been conclusively linked to a particular ROS source or studied in epithelial barriers, we will present here only results that identified the enzyme involved, were obtained by multiple approaches and if possible *in vivo*. In the GIT, superoxide produced by NOX1 controls colon epithelial cell proliferation and migration, in part by enhancing FAK phosphorylation due to oxidative inactivation of the tyrosine phosphatases LMW-PTP and SHP-2 that will initiate focal adhesion turnover, accelerate cell migration, and improve wound closure (81–85). Others reported NOX1-mediated oxidation of nucleoredoxin that released suppression of the canonical WNT-β-catenin pathway, resulting in transcription of growth promoting genes (86, 87). In airway epithelial cells DUOX was localized at the leading edge of migrating cells, augmenting wound healing (88). DUOX1 generated H_2_O_2_ regulated cell migration and epithelial repair in cells and in a naphthalene airway injury model by promoting epidermal growth factor receptor (EGFR)-STAT3 signaling (89, 90).

An important part of the physical gut barrier is the protective mucus layer, either single-layered in the small intestine and pulmonary tract, or 2-layered with a dense, impenetrable layer followed by a loose layer in the colon. Mucus is secreted and continuously renewed by goblet cells and is composed of mainly MUC2 mucin in the intestine, MUC1, MUC5AC and MUC6 mucins in the stomach, and MUC5AC and MUC5B mucins in the lung epithelium (91). NADPH oxidase generated H_2_O_2_ has been linked to intestinal mucin granule release by pathways involving endocytosis, autophagy, and NLRP6 signaling (92, 93). A severe colonic phenotype with partial loss of the dense mucus layer was only observed in mice with combined inactivation of Nox1-3 but not in single oxidase knockout mice (94), likely due to *in vivo* compensation by another oxidase (or ROS source) or by H_2_O_2_ producing lactobacilli colonizing the mucus layer. In the airways EGFR and IL-13 signaling promoted mucin gene induction (MUC5AC, CLCA1) *via* DUOX1 (95, 96), while others reported a TLR5-DUOX2 pathway regulating MUC5AC expression in nasal epithelium (97).

The structural reinforcement of the intestinal barrier is accompanied by chemical and immunological secretions. Immunological secretions are immunoglobulin A (sIgA) and various other antimicrobial factors including lysozyme, regenerating islet-derived proteins 3, and cationic peptides such as cathelicidins or defensins. Cationic peptides range from α-helical (e.g., LL-37) to β-sheet (e.g., β-defensins) conformation or random-coil structures and exert their activity by bacterial membrane disruption and immunomodulatory effects on host cells (98, 99). While their synthesis or secretion has not been linked to a ROS source, the proper folding of some bioactive cysteine-rich peptides such as β-defensin 3 require the oxidative formation of several disulfide bridges (100). Lipocalin-2 (LCN-2), secreted by intestinal epithelial cells or released from neutrophil secondary granules, is often used as a biomarker of inflammatory processes, but also limits bacterial growth by binding iron-loaded siderophores (101, 102). A recent report connected LCN-2 expression *via* atypical IκBζ-dependent gene transcription to NOX1 activity in colonic epithelial cells and mice (103).

Mucosal barriers generate and release chemical compounds such as superoxide, H_2_O_2_, nitric oxide and peroxynitrite when pathogens or danger-associated molecules trigger host cell responses. These chemicals may also be released constitutively at low concentrations as repellent against commensal communities, promoting a mutualistic host-microbiota relationship essential for immunity (21, 104). This is supported by reports that enteric bacteria regulate NADPH oxidase activity by relatively undefined pathways (85, 105, 106), and vice versa that ROS generated by mitochondria or epithelial cells control the microbial population, thereby limiting the access of the microbiota to the immune compartment (19, 107). This scenario likely takes place in the small intestine with its loose, single mucus layer and may involve the oxidase DUOX2. DUOX2 expression is upregulated by microbiota, and its localization at the apical surface of villi is ideally suited for H_2_O_2_ release, thereby reinforcing the separation of host and microbial communities (19). Additionally, peroxynitrite generated by the combined activation of NOX1 and NOS2 may participate in the control of ileal homeostasis (108, 109). Similarly, H_2_O_2_ release by DUOX1/DUOX2 and subsequent lactoperoxidase-mediated conversion to secondary oxidants (i.e., HOSCN) may repel bacteria in the airways (110). Influenza A virus (IAV) infection stimulated H_2_O_2_ release from air-liquid interface cultured primary human airway epithelial cells in a calcium/flavoenzyme dependent manner, suggesting DUOX activation (111).

Chemical messengers are ideally suited to relay signals from the host to the microbiota and vice versa. This interkingdom communication will shape microbiota diversity and composition and provides a stimulus for homeostatic barrier host responses. While aberrant ROS production in inflammatory disease will increase the abundance of certain bacterial communities and may lower overall diversity, decreased levels or loss of NADPH oxidase-generated ROS, as observed in certain patient populations, will also favor dysbiosis, indicating that a tightly balanced mucosa-associated microenvironment is indispensable for gut homeostasis. While CGD is mainly associated with recurrent life-threatening infections, 40-50% of these patients will develop inflammatory bowel disease with some features of Crohn’s disease. When comparing the microbiota of CGD patients to healthy individuals an increased abundance of mucus foraging *Ruminococcus gnavus* was noted (112). This study included only a limited number of patients (10 individuals) and might be skewed due to the required antibiotic maintenance of these patients. In a CGD mouse model (i.e., p47^phox^ deficiency) the microbiome signature was altered by high abundance of *Akkermansia muciniphila* (113). This mucolytic bacterium is considered a beneficial probiotic, but can also disturb mucus homeostasis in the host, thereby acting as a pathobiont or aggravating inflammation induced by intestinal pathogens (114–116). In the ileum of Nox1 knockout mice increased abundance of Bifidobacteria and Turicibacter was detected (117). Global inactivation of the oxidases Nox1-3 in mice (*Cyba^nmf333^*) resulted in increased abundance of Proteobacteria, Ruminococcaceae and *Mucispirillum schaedleri* (94). An increase of *M. schaedleri* was also noted in mice with combined Nod2/Nox2 deficiency (118). This mucus dwelling bacterium interfered with *Salmonella enterica* serovar Typhimurium virulence factor expression and decreased pathogen invasion in wildtype mice (119), but it also promoted inflammation in Nod2/Nox2 double knockout mice. Inactivation of all four murine Nox enzymes by global or intestinal epithelium restricted deletion of the essential Nox partner protein p22^phox^ (*Cyba^-/-^*, *Cyba^Vil-cre^*) induced compensatory upregulation of H_2_O_2_ producing lactobacilli as a host protective, mutualistically beneficial mechanism (120). These results in mice suggest that H_2_O_2_ generation, mucus changes and overgrowth of mucus-dwelling or mucolytic bacteria are closely linked. Thus, ROS maintain and protect the stable microenvironment required for balanced and interconnected host-commensal cooperation and homeostatic barrier function.

### Specialized Roles of Oxidants in Innate Immune Cells

#### Macrophage Polarization

The versatile role of ROS in regulating the intracellular signalosome in a constantly evolving microenvironment underpins their role in promoting polarization of immune cell populations and supporting specialized functions. A pertinent example is altering the polarization and activation state of macrophages in the broad categories of M1 (classical) and M2 (alternative), categorized by a proinflammatory, host defense connected phenotype versus an anti-inflammatory, tissue remodeling phenotype (121). NOX2 is expressed in M1 and M2 macrophages and regulates the transition between phenotypes in a context dependent manner. Nox2 deficient bone marrow derived macrophages (BMDM) upregulated STAT3 activation and anti-inflammatory cytokine expression (i.e., IL-10), while others reported no differences between wildtype and Nox2 knockout M2 macrophages (122, 123). Conflicting results in the literature stem likely from the range of protocols used for macrophage differentiation (e.g., conditioned media versus recombinant GM-CSF or M-CSF for various time periods, LPS content of fetal bovine serum) and the inclusion or omission of further Th1 or Th2 type polarization. M1 polarized BMDM express not only Nox2 but also Nox1 (124). When M-CSF differentiated BMDM or peritoneal macrophages derived from wildtype, Nox1, Nox2 or Nox1/Nox2 knockout mice were further polarized by LPS+IFNγ or IL-4+IL-10, only Nox1/Nox2 deficient macrophages exhibited reduced M2-type polarization while M1 polarization and pro-inflammatory functions were preserved (123). Expression of Nox4 in macrophages and its downstream effects seem to be determined by the microenvironment. Using a similar M1/M2 polarization protocol as described above Helfinger and coworkers observed in Nox4 deficient mouse macrophages amplification of the M1 phenotype, suggesting an anti-inflammatory role for Nox4 (125). On the other hand, NOX4 expression was induced in human monocyte-derived macrophages by low-density lipoprotein (OxLDL) that stimulates a pro-inflammatory response (126), and in profibrotic M2 alveolar macrophages (127). A mechanistically poorly defined link between NOX4 and mROS has been proposed (i.e., ROS-induced ROS) that could play a role in conferring the divergent metabolic signatures observed in M1 versus M2 macrophage polarization (127–131). H_2_O_2_-mediated intercellular communication between various innate immune cells can also drive macrophage skewing. In vivo phenotypic conversion of pro-inflammatory to pro-resolving macrophages was dependent on neutrophil Nox2 and H_2_O_2_ generation, further confirming that activated neutrophils contribute to resolution of inflammation (132–135). While less characterized, neutrophils can, analogous to macrophages, polarize towards distinct phenotypes. N1 and N2 neutrophil populations are mainly defined by their functional phenotype with N1 considered pro-inflammatory and anti-tumorigenic, and N2 pro-tumorigenic. A role for ROS in this phenotypic conversion has not yet been defined.

#### Dendritic Cell Function

Dendritic (DC) cells are antigen-presenting cells that are essential for inducing naïve T cell activation and effector differentiation. After taking up antigens and microbes by phagocytosis or endocytosis DCs generate MHC peptide complexes. For activation of CD4 + T helper cells, antigens are degraded in lysosomes for MHC class II presentation, while activation of CD8 + cytotoxic T cells requires that antigens are presented in MHC class I by cross-presentation that occurs *via* lysosomal and cytosolic pathways. Interaction of mature DCs with T cells takes place in secondary lymphoid organs such as lymph nodes, spleen, and Peyer’s patches, initiating adaptive immune responses (136). Superoxide production by NOX2 is an intricate part of antigen processing, generation of MHC-peptide complexes and cross-presentation. Work by Amigorena and coworkers revealed that active NOX2 regulates the phagosomal pH in DCs by sustained superoxide generation, maintaining it close to the optimal pH 7.4 despite of proton import by V-ATPase. In DCs derived from CGD patients or CGD mice the phagosomal pH acidified and the cross-presentation of antigens was impaired (137, 138). In addition, NOX2 activity preserved antigens from proteolysis by inhibiting endosomal and lysosomal proteases via oxidation (139). Recent work connected NOX2 to antigen release from the endocytic lumen into the cytosol during cross-presentation. NOX2-derived superoxide caused lipid peroxidation, thereby disrupting membranes to enable antigen leakage from endosomes, a process that was impaired in DCs derived from CGD patients (140). Further study of a specialized subset of DCs, plasmacytoid DCs (pDCs), distinguished from conventional DCs by morphology and function, revealed that in this cell type antigen protection and cross-presentation is Nox1- and Nox2-independent. Here, mROS generated by pDCs after TLR7 stimulation promoted antigen cross-presentation and the capacity to trigger CD8+ T cells responses, as well as facilitating additional functions such as IFN-α production (141). Antigen presentation is also linked to macroautophagy that serves not only as mechanism for nutrient recycling and host defense against intracellular bacteria, but also for presenting MHC II molecules to CD4+ T cells. Recruitment of autophagy proteins to phagosomal membranes occurs when antigens are recognized by immune receptors such as TLRs, FcRs or dectin-1, resulting in LC3-associated phagocytosis (LAP) (142, 143). LAP requires NOX2-generated superoxide for recruitment and lipidation of the autophagy protein Atg8/LC3 to phagosomes, and fungal antigen storage in innate immune cells was compromised in CGD patients (144, 145). NOX2 activity was also involved in antimicrobial LAP in endothelial cells (146). Autophagy processes involved in cellular maintenance seem to be coupled to oxidase-derived ROS in many cell types including to NOX4 in cardiomyocytes and to a NOX family member in goblet cells (92, 94, 147), but putative ROS source(s) facilitating pathogen uptake due to LAP or other processes in epithelial cells, an important consideration for mucosal barrier host defense, have not yet been defined.

### Host Defense and Oxidants

Respiratory and intestinal barrier epithelia are the first line of defense against airborne or foodborne pathogens. When the initial protective mechanisms (e.g., colonization resistance, antimicrobial secretions, mucus layer) have failed, epithelial sensing pathways will be activated. In the mucosal compartment, sensing of conserved microbial motifs by epithelial cells induces ROS production due to upregulation and/or activation of mucosal NADPH oxidases (e.g., DUOX1/2 in airways, NOX1/DUOX2 in GIT) that participate in the antimicrobial response (97, 110, 148–150). Transient or stable expression of the NOX1 or DUOX2 complex in several epithelial cell lines decreased pathogen attachment and invasion without affecting bacterial viability (151, 152). NOX1 or DUOX2 mediated H_2_O_2_ release interfered with the pathogenicity of bacteria by inducing irreversible oxidative modifications in bacterial enzymes and proteins essential for maintaining virulence factor synthesis (22, 23). Duox inactivation in mice, achieved by deleting the essential partner protein Duoxa, increased gastric colonization with *Helicobacter felis*, reflecting the protective effect of H_2_O_2_ in host defense (153). Infection of these mice with *Salmonella* Typhimurium augmented systemic dissemination of the pathogen, suggesting that Duox is an integral component of the protective mucosal barrier in the small intestine (19). In murine airways Duox silencing increased the Influenza A virus (IAV) load by interfering with viral replication, at least in part by affecting the nuclear splicing machinery and assembly of virions (111). Recent studies in DUOX2 knockdown primary human nasal epithelial cells and in Duox2 inactivated mutant mice linked this oxidase isoform further to IAV host defense (154). Nox1 has also been connected to host protection in IAV infection as Nox1 deficiency resulted in increased chemokine and proinflammatory cytokine generation, and extensive 3-nitrotyrosine modification of murine lung tissue (155). In IAV infected mouse macrophages Nox2-derived endosomal ROS caused suppression of TLR7-dependent antiviral cytokines, suggesting distinct roles of phagocyte Nox2 (i.e. detrimental) in contrast to a beneficial role of Nox1 during IAV infection (156).

Pathogen recognition by epithelial cells induces chemokine production, leading to the recruitment of neutrophils and macrophages to the site of injury. Innate immune cells engage in proteolytic and oxidative killing of pathogens, mainly mediated by secondary reactive species such as hypochlorous acid and peroxynitrite that rely on NOX2, mitochondria, myeloperoxidase and iNOS (157–159). Superoxide generated by NOX2 is essential for host protection against pathogens, as mutational inactivation of NOX2 complex subunits, a characteristic feature of CGD patients, profoundly impedes the ability to clear certain bacterial and fungal infections (160). Studies using murine CGD hosts have advanced our understanding of the mechanisms associated with host protection by Nox2 that seem to depend on the pathogen and the microenvironment encountered. For instance, intestinal colonization by *Salmonella* Typhimurium was increased in streptomycin pretreated Nox2 deficient mice (i.e., *Cybb^-/-^*) (161). Conversely, these mice were not more susceptible to infection with the murine pathogen *Citrobacter rodentium* (120). Superoxide produced by Nox2 played only a minor role in protection against *Mycobacterium tuberculosis* (Mtb) infection in contrast to reactive nitrogen species (RNS)-associated immunity, which seems preponderant in the control of murine lung infection with Mtb (162–165). Evasion of ROS-mediated killing (mROS and Nox2) was recently associated with Mtb virulence factor secretion and macrophage fatty acid catabolism, thereby improving mycobacterial survival in macrophages (166, 167).

These observations support the idea of a complex interaction between antimicrobial ROS, environmental factors, and virulence mechanisms employed by pathogens (e.g., detoxification enzymes), all impacting infection outcome and host protection (168). Redox-dependent mechanisms triggered by pathogen recognition in innate immune cells include the pathogen itself (e.g., size, type), and driving superoxide production in the appropriate subcellular compartment (e.g., phagosome, endosome, extracellular environment) by a specific source (e.g., NOX2, mitochondria) (169, 170). Upon pathogen uptake the phagosome is the main compartment where primary and secondary ROS accumulate in high concentrations. Assembly and activation of NOX2 at the phagosome membrane triggers the so-called ‘phagocyte oxidative burst’ that together with proteases supports killing of bacterial and fungal pathogens. In addition to phagosomal NOX2 activity, bactericidal ROS generation in the phagosome is maximized by mitochondria, with both ROS sources cooperating in pathogen killing (171). Mitochondria are recruited to the phagosome of infected macrophages, where they deliver mitochondria-derived effector molecules, including mROS in mitochondria-derived vesicles, to support intraphagosomal killing of pathogens (68, 172–174). Mitochondria mobility to the phagosome is promoted by the protein kinases Mst1/2. Upon phagocytosis, TLR-mediated signaling activates Mst1/2, triggering a TRAF6-Rac signaling axis involved in cytoskeletal rearrangements (172). The NOX2 complex localizes also to the plasma membrane, where it releases superoxide into the extracellular space in response to microbial patterns or large microorganisms that cannot be classically engulfed by phagocytic cells.

Neutrophils employ as additional host defense mechanism the release of nuclear chromatin, forming neutrophil extracellular traps (NETs). NETs act as defense mechanism against pathogens *via* entrapment and antimicrobial activity (175, 176). The sequence of events for NET formation is not completely defined (177), but it appears that NET release can be triggered in a ROS dependent or independent manner according to the pathogen or stimulus encountered (178–180). Neutrophils derived from CGD patients show an impairment in NET formation and ineffective control of *Aspergillus* species in the airways, leading to invasive aspergillosis. The importance of a functional NOX2 complex in restricting *A. nidulans* conidia and hyphae by NET formation was confirmed in a CGD patient undergoing gene therapy (181). The real-life situation of the severely compromised host immunity in CGD emphasizes the importance of ROS in host defense and serves as a reminder that certain disorders or disturbed mucosal environments may greatly benefit from improving oxidant production.

## Improving Ros Levels as Therapeutic Intervention

**Figure 1:**
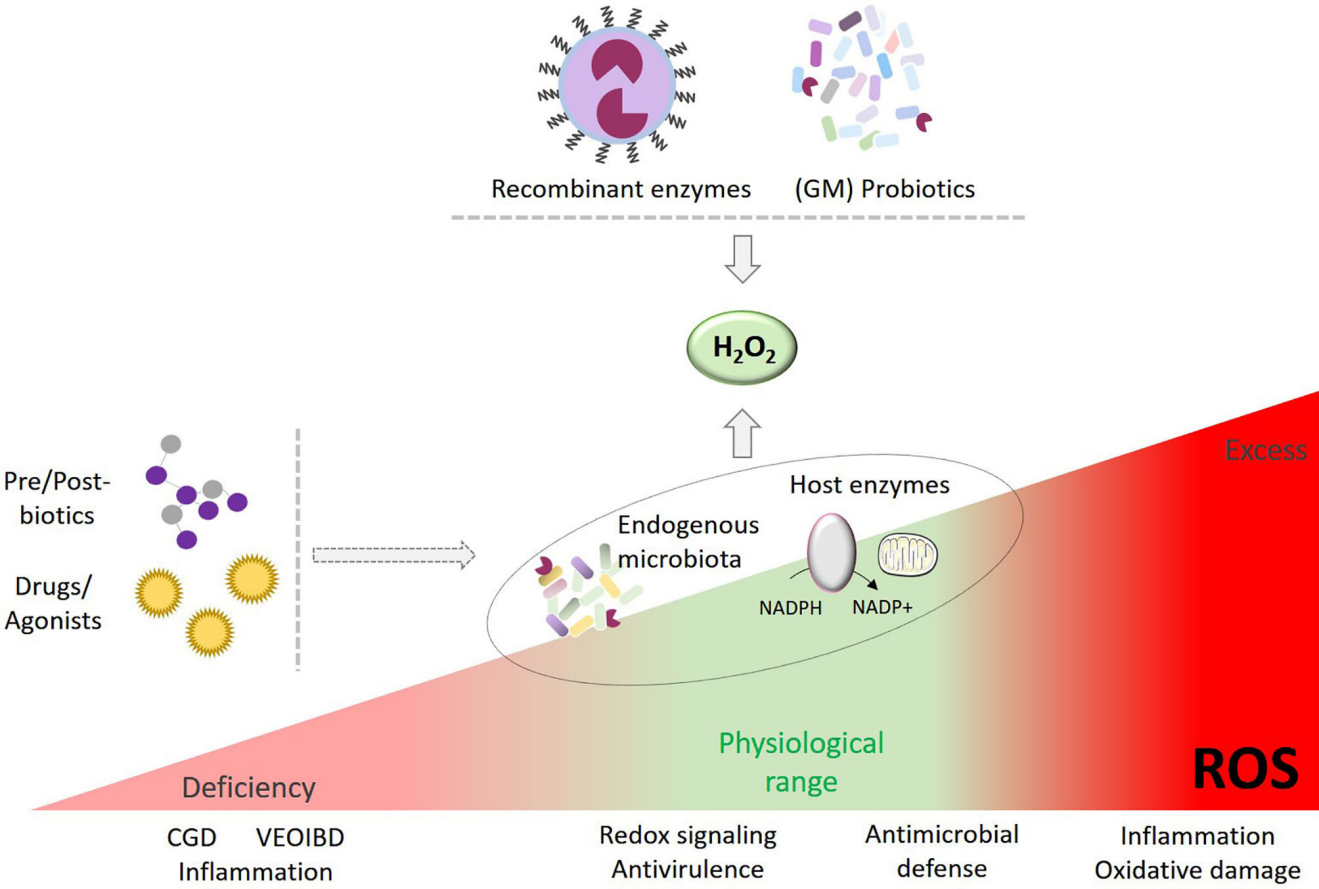
Enhancing H_2_O_2_ as therapeutic intervention. Strategies to restore or improve ROS in a controlled manner may include a) administration of selected probiotics or genetically-modified (GM) bacteria producing H_2_O_2_, b) innovative drug technology delivering recombinant H_2_O_2_ generators to a target area, c) pre-/post-biotics and d) drugs and agonists modulating ROS-generating host enzymes or endogenous microbiota to augment H_2_O_2_ production.

In pathological conditions ROS have been linked to ‘oxidative stress’ for decades. Oxidative stress is often retrospectively inferred when observing oxidatively modified proteins, lipids and DNA, yet without definite identification of the ROS generating enzyme(s) involved or how the damaging sequence of events was initiated. Moreover, several disease-causing genetic variants and disease-associated genetic risk factors have shed light on the harmful consequences of insufficient ROS production for H_2_O_2_-induced oxidative modifications, redox signaling and host defense (e.g., CGD, hypothyroidism, inflammatory bowel disease) (33). To date most strategies targeting oxidative stress (i.e., antioxidant therapy) are based on the delivery of ROS scavengers, ROS converting or degrading compounds or on non-selective enzyme inhibitors to protect tissues and organs, but clinical results of antioxidant treatments in inflammatory diseases have so far been disappointing (182–184). More recently, treatments aimed at generating excessive concentrations of ROS or at inhibiting cellular antioxidant defense systems at a designated target location (i.e., prooxidant therapy) have been developed to promote cancer cell death or antimicrobial killing through oxidative damage (185, 186). In contrast, ROS/redox-modulating therapies take a different, nontraditional approach. The goal of this strategy is a middle path intended to restore physiological levels of primary ROS (superoxide, H_2_O_2_) or, in certain circumstances, to moderately augment beneficial oxidants. This approach will require innovations in pharmaceutical drug technology such as nanotechnology and smart materials (e.g., liposomes, hydrogels, nanocarriers) enabling controlled delivery of therapeutic agents (e.g., ROS producing or converting systems) to a target site due to utilization of stimulus-sensitive materials (187–190). In some settings ROS deficiencies may be partially compensated by pharmacological manipulation of endogenous oxidant producing enzymes. While still in their infancy science-based fine-tuning and balancing oxidant therapies are poised to deliver prophylactic and remedial benefits, namely improving homeostasis and host defense. This is particularly relevant for primary ROS with their essential role in redox signaling and dedicated immune functions. Here we summarize concepts for restoring physiological levels of beneficial oxygen-derived species in terms of technological approaches and therapeutic applications (**Figure 1**).

### Established and Emerging Strategies for Oxidant Generation

#### Use of Hydrogen Peroxide

In the past, external application of H_2_O_2_ has been used for medicinal purposes (191, 192). Although high concentrations of H_2_O_2_ have well-known antiseptic properties, tissue damage may occur. On the other hand, application of lower concentrations of H_2_O_2_ (0.15-1.25 µmoles/wound) improved the rate of wound closure in wildtype mice and in mice deficient in Nox2-derived superoxide, thereby accelerating tissue regeneration (193, 194). Topical application of micromolar H_2_O_2_ promoted the phosphorylation of tyrosine residues in FAK that supported an angiogenic response *via* vascular endothelial growth factor (VEGF) signaling. Removal of H_2_O_2_ by addition of the H_2_O_2_ degrading enzyme catalase delayed wound healing and impeded angiogenesis (193). While these experiments may suggest direct exposure of skin to H_2_O_2_ as wound healing treatment, the therapeutic window is too narrow for safe and efficient tissue restitution. Further, internal administration of H_2_O_2_ solutions poses serious health risks and should not be promoted. To achieve a health benefit, technologies permitting modified release of a standardized dose of nanomolar H_2_O_2_ to specific target areas will be required. Strategies promoting controlled H_2_O_2_ delivery may include the utilization of nano systems, diffusible molecules, and stimulus-sensitive compounds (195–198). Stimulus-responsive materials are particularly attractive to target specific disease-associated microenvironments such as changes in pH due to inflammation (189, 199, 200). In accord, one can imagine the development of redox-sensitive materials actively releasing appropriate oxidants until a certain threshold is achieved and the system self-inactivates. Moreover, the vehicle strategy is important. For instance, hydrogels are commonly used as drug carriers for clinical use, owing to their tunable mechanistic and physicochemical properties (201). Controlled and continuous release of H_2_O_2_ was achieved with an *in situ* forming hydrogel that can be used as antimicrobial wound dressing (202).

#### Lactobacillus as H_2_O_2_ Source

Lactobacilli, widely used probiotic bacteria that belong to the gastrointestinal and vaginal microbiota (203), are the microbial prototype for controlled release of nanomolar H_2_O_2_. Many *Lactobacillus* strains utilize oxygen to generate H_2_O_2_ by various enzymes including flavin reductase (nfr), NADH oxidase (nox) or pyruvate oxidase (pox) (204–207). Lactobacilli colonizing the mucus layer are close enough to the oxygen gradient emanating from host epithelial barrier cells (up to 3% O_2_) to secrete nanomolar H_2_O_2_ continually (208–210). A recent report linked *Lactobacillus* generated H_2_O_2_ to increased colonization resistance, protecting the host from *C. rodentium* infection (120). A decline in host-derived H_2_O_2_ due to genetic deletion or loss-of-function mutations in NADPH oxidases gives rise to microbiota dysbiosis, which is likely niche-driven and tied to changes in the intestinal microenvironment. Thus, manipulation of the microbiota with lactobacilli constitutes a potential strategy to promote microbiota diversity for improved colonization resistance. This approach can be extended to the airways colonized by their unique microbiota. Intranasal administration of probiotics (including various *Lactobacillus* strains) was recently proposed to modulate local immunity and epithelial barrier function (211, 212). In addition to living lactobacilli, administration of prebiotics to selectively stimulate the growth or activity of resident lactobacilli may improve the barrier redox environment and stimulate host immunity. For example, abundance of lactobacilli seems to increase following the consumption of galacto-oligosaccharides, a common prebiotic generated from the decomposition of lactose, with reported beneficial effects on immune function (203, 213).

Early findings that H_2_O_2_ promotes cell migration match with the positive effects of lactobacilli on wound healing. Lactobacilli secrete not only H_2_O_2_ but numerous other compounds such as lactic acid and bacteriocins that improve the wound environment. Nevertheless, the ability of *L. johnsonii* to generate H_2_O_2_ was directly linked to accelerated recovery and tissue restitution in murine colitis (210). Transformation of lactobacilli to express exogenous proteins can further improve their wound healing capacity as demonstrated recently for *L. reuteri* secreting CXCL12 (214). Lactobacilli may also release yet undefined compounds that trigger intracellular ROS generation by host cells, for example by the oxidase Nox1, to improve mucosal repair (85, 105). Bacteria or microbial-derived products can drive expression of ROS-generating enzymes in the host, likely associated with increased H_2_O_2_ levels. Ileal colonization with commensal segmented filamentous bacteria (SFB) increased expression of the oxidase Duox2 (19). Intraperitoneal administration of SFB-derived flagellin was sufficient to upregulate Duox2 in the small intestine, suggesting that microbial regulation of Duox2 expression may be TLR5 dependent (215).

Most studies linking lactobacilli to antimicrobial defense, mucosal healing and microbiota modification have been conducted in mice. In clinical trials the outcome of probiotic therapy in IBD has been mixed (216–219). Possible explanations range from poor standardization and viability of bacteria to insufficient colonization due to the human intestinal environment including the mucus layer, as well as to differences between mouse and human physiology such as the presence of a *Lactobacillus* reservoir in the murine forestomach. Another confining factor is using living organisms, which can lead to systemic bacterial dissemination. Several reports outline the risk for *Lactobacillus* bacteremia and septicemia in immunocompromised patients, after surgical procedures and in colitis patients (220–223). Even in mice septicemia was observed when either the orally administered dose of *L. johnsonii* was increased ten-fold or *L. johnsonii* overproducing H_2_O_2_ was administered, indicating that H_2_O_2_ production should not exceed an optimal physiological range for health benefit (210). Current developments in the field are focused on standardization, design of consortia and recombinant probiotics, but other inventive strategies as outlined below could address some shortcomings of probiotics as H_2_O_2_ generators.

#### Application of Recombinant Enzymes

Recombinant enzymes involved in ROS generation or conversion can be used to modulate the oxidative microenvironment. The enzyme superoxide dismutase (SOD) catalyzes the dismutation of superoxide into oxygen and H_2_O_2_. Several isoforms of this enzyme are expressed in both mammalian and bacterial hosts, where they serve as part of the protective antioxidant system. SOD-mediated superoxide conversion limits the generation of secondary, highly reactive oxygen and nitrogen metabolites (e.g., hydroxyl radical, peroxynitrite) that can cause irreversible modification of proteins, lipids or DNA (224, 225). Therefore, SOD-based applications have been mainly used as antioxidant strategy aiming to decrease secondary ROS levels for prevention of inflammatory diseases. A more universal utilization of SOD as physiological H_2_O_2_ source at mucosal surfaces is compromised by its mode of action, including reliance on inflamed conditions, in conjunction with unattainable dosing standardization, and poor enzymatic stability (226).

Many oxidoreductases generate H_2_O_2_ as a byproduct of their intended enzymatic reaction including carbohydrate oxidases [e.g., glucose oxidase, galactose oxidase and many others (227)], cholesterol oxidase (228), alcohol oxidase (229) and D-amino acid oxidase (230). We will discuss here as example glucose oxidase (GOx), a prominent representative of carbohydrate oxidases that catalyzes the oxidation of β-D-glucose to D-glucono-1,5-lactone with further hydrolysis to gluconic acid. GOx contains the flavin adenine dinucleotide (FAD) cofactor as initial electron acceptor, which undergoes reduction to FADH2, followed by FADH2 oxidation by molecular oxygen, and reduction of oxygen to H_2_O_2_ (231). GOx is found predominantly in fungi (i.e., *Aspergillus* and *Penicillium* spp) and its high specificity for glucose renders the enzymatic reaction an attractive tool for monitoring blood glucose levels. Various GOx-based biosensors have been developed for glucose monitoring, sometimes in diabetes therapy in conjunction with insulin release (232, 233). Stability of the enzyme is important for these applications, and thus technology advancements have resulted in genetically engineered GOx modifications in combination with encapsulation strategies for improved long-term enzymatic activity (234). Dermatological applications of GOx as tunable H_2_O_2_ generator may hold some promise for therapy. Incorporation of GOx in a collagen dressing improved wound healing and tissue regeneration of rodent skin, presumably due to sustained release of H_2_O_2_, although oxidant measurements were not provided. This GOx treatment induced an antioxidant response by the host tissue with upregulation of SOD and catalase (235). GOx is naturally present in honey at low concentrations where it has been studied for its dual function, namely for aiding wound healing and as antimicrobial agent. A medicinal honey (SurgihoneyRO™) with enhanced antimicrobial activity is on the market as a wound antiseptic dressing (236, 237). Due to its GRAS (generally recognized as safe) FDA status GOx is used by the food industry as stabilizing and antibacterial agent (238).

The GRAS status supports considering GOx for internal applications such as hydrogels or oral administration of the enzyme. A gelatin hydrogel incorporating glucose and varying GOx concentrations released micromolar H_2_O_2_ for 24-48 hours in a controlled manner and improved the proliferation of cultured endothelial cells. This effect was accompanied by a transient increase of intracellular ROS (measured as DCFH-DA signal) as well as enhanced neovascularization in a CAM model, indicating modulation of redox signaling by GOx-generated H_2_O_2_ (239, 240). For oral administration, the protection of GOx’s enzymatic activity can be achieved by microencapsulation. Diet delivered glucose might be sufficient for GOx-mediated H_2_O_2_ generation in the small intestine, while supplementation of the GOx drug carrier with compartmentalized glucose will be necessary for affecting the large intestine. Targeted, controlled delivery of GOx/glucose presents a promising opportunity to modulate intestinal redox signaling, mucosal healing and immune defense while limiting oxidative damage. Therapeutic applications of GOx/glucose or similar H_2_O_2_ generators will partially mimic the current use of lactobacilli, albeit with superior standardization, more limited dependence on the barrier microenvironment and superior safety in vulnerable patient populations. An additional benefit of the GOx/glucose reaction is the generation of the prebiotic gluconic acid as a secondary reaction product, which stimulates butyrate production in the intestine (241). Examples for potential treatment modalities are gut health improvements (e.g., barrier reinforcement, microbiota diversity), accelerated tissue restitution after injury in patients with intestinal inflammatory diseases, or prophylactic long-term modification of the intestinal environment in patients with insufficient H_2_O_2_ generation. Prime examples for the last point are certain patient populations in the categories very early onset IBD (VEO-IBD), CGD-IBD or IBD linked to variants upstream of ROS-generating enzymes (21, 242–244). Variants in genes encoding components of NOX enzymes including NOX1, NOX2 and DUOX2 confer susceptibility to VEO-IBD (152, 245–248), while NOX2 complex variants associated with CGD due to absent or minimal output of superoxide manifest in 40-50% of CGD patients as CGD-associated IBD. Functional evaluation of NOX1 and DUOX2 patient variants in model systems revealed decreased ROS production and impaired antimicrobial defense (152, 246, 247). The compromised immune defense of these hosts in combination with the ensuing dysbiotic microbiota affects not only the overall colonization resistance but also more specialized immune defense mechanisms. Diffusion of nanomolar H_2_O_2_ into extracellular pathogens is not bactericidal but can downregulate virulence factors. Nanomolar H_2_O_2_ blocked bacterial phosphotyrosine signaling required for virulence factor synthesis by irreversibly modifying tyrosine phosphorylated enzymes and proteins (22, 23). In addition, nanomolar H_2_O_2_ inhibited LEE pathogenicity island regulation in enteropathogenic bacteria (*C. rodentium*) due to impeded expression of the major transcriptional regulator *ler*, which was associated with lower pathogen colonization and improved recovery of mice (120). While the precise molecular mechanism of LEE downregulation has not yet been resolved, these studies hold great promise for H_2_O_2_-mediated interference in enteric bacterial infections. Supplying nanomolar levels of H_2_O_2_ *via* GOx/glucose or similar means may provide multiple benefits including host protection, reinforcement of the intestinal barrier and mucosal healing.

#### Stimulation of Endogenous ROS Sources

Instead of providing ROS by an exogenous source, agonists or drugs that stimulate expression and/or activation of a ROS-generating enzyme or alter mitochondrial function can be used to improve immune responses. Currently this area is dominated by improving innate immune cell-derived superoxide, but one can envision that future applications will be directed at epithelial superoxide/H_2_O_2_-producing enzymes. Neutrophils and macrophages are crucial for pathogen defense with NOX2-derived superoxide playing a key role in this process. Enhancing the neutrophil activation status, and in particular the oxidative burst, will not only be desirable in infections but also in inflammatory disorders (249–251). Activating NOX2 can be achieved by targeting upstream pathways. One approach focusses on developing formyl peptide receptor (FPR1, FPR2) agonists selectively binding to a receptor conformation that will favor one signaling output over another (i.e. biased signaling) without accelerating receptor desensitization (249). In neutrophils FPRs regulate directional migration, secretion of inflammatory mediators and superoxide generation by NOX2. While FPRs are usually triggered by microbial derived formylated peptides, endogenous proteins or lipopeptides, small compounds and peptides acting as exogenous FPR agonists have been identified. Examples are the FPR1 agonist RE-04-001, FPR2 agonists BMS-986235 and Act-389949, and dual FPR1/2 agonists such as compound 17b (252–255). Biased FPR agonists causing functional selectivity in neutrophil responses, namely either inducing chemotaxis or superoxide production, can be used as NOX2 activators (252, 256). Nox2 activity has been associated with the resolution of inflammation in colitis (133), and the pro-resolving Fpr2 agonist Annexin-A1 accelerated wound healing of the murine colonic epithelium *via* the oxidase Nox1 (81).

Another approach is using the cytokine granulocyte-macrophage colony-stimulating factor (GM-CSF) to augment NOX2 derived superoxide production by acting as priming agent in neutrophils while elevating the phagosomal pH in macrophages (257–259). The efficacy of GM-CSF administration as immunostimulatory adjuvant has been evaluated in clinical trials. In a randomized trial involving critically ill patients, subcutaneous injections with GM-CSF increased neutrophil phagocytic capacity but did not improve superoxide production (EudraCT 2011-005815-10), while an earlier trial using intravenous delivery of GM-CSF had reported statistically significant enhancement of neutrophil superoxide generation (260). GM-CSF has also been assessed in the context of CGD patients. While reconstitution of the oxidative burst in isolated neutrophils was not achieved, as expected for a disease caused by genetic mutations, GM-CSF has shown some promise in treating CGD-IBD by undefined mechanism(s) (261–263). In non-CGD patients biased targeting of NOX2 activating pathways may hold promise, and analogous strategies should be feasible for development of activators of other NOX/DUOX enzymes or of other ROS sources.

#### Generation of Compensatory ROS

Therapeutically enhancing ROS production in CGD or VEO-IBD patients is imperative for antimicrobial defense, for resolution of inflammation and for repair processes. In these patients gene editing, administration of exogenous ROS generators or stimulation of other endogenous ROS sources are the only options to improve their ROS status. Kuhns and coworkers showed that modest residual superoxide production is sufficient to prevent life-threatening infections in CGD patients (264). Partial restoration of macrophage and dendritic cell superoxide in functional *Ncf1* rescue mice was adequate to dampen hyperinflammation (265). These considerations are the basis for screening and identifying compounds capable of compensating partially for NOX2 enzyme activity.

The first compound in this class is the peroxisome proliferator-activated receptor γ (PPARγ) agonist pioglitazone, a drug approved for Type 2 diabetes. As proof of concept, pioglitazone treatment of cultured CGD patient-derived monocytes or of Nox2 deficient mice increased mROS production and subsequently the bactericidal capacity of immune cells (266). Pioglitazone and the related rosiglitazone induced mROS and NETs in CGD neutrophils (267). Monocytes from CGD patients and Nox2 deficient macrophages were impaired in PPARγ signaling, impacting their efferocytosis function which was restored by pioglitazone treatment (268, 269). Increased mROS generation and restoration of efferocytosis were also observed in monocytes isolated from two CGD patients after 30 days of oral pioglitazone treatment, supporting the effectiveness of pioglitazone therapy for superoxide production and improved immune cell function (269). A clinical study reported the effects of daily pioglitazone treatment in an infant with CGD. Using dihydrorhodamine (DHR) fluorescence as readout for ROS an increase in DHR positive granulocytes after 25 days of treatment was observed. The increase in the phorbol ester stimulated DHR signal was relatively low in comparison with healthy donor cells but it was maintained over several weeks, and the clinical condition of the patient progressively improved (270). Efficacy and safety of pioglitazone was assessed in a clinical trial enrolling CGD patients with severe infection. Phase 2 of the study was terminated when the DHR fluorescence signal did not improve in neutrophils after 90 days of treatment with pioglitazone (clinicaltrials.gov NCT03080480). Long-term administration of pioglitazone did not lead to drug-related adverse effects or exacerbation of infection (271). The variation in outcomes might be attributable to different pioglitazone dosage, or to the often required combination therapy with antibiotics and/or interferon IFN-γ. Further studies are necessary to determine if pioglitazone will provide a therapeutic option for CGD patients, for example as adjuvant therapy in severe bacterial infections or as prophylaxis. At this point only hematopoietic stem cell transplantation, gene therapy or gene editing with CRISPR-Cas9 offer a cure for CGD (272).

### Future Directions

The field of redox medicine has blossomed over the last decade, but further progress will depend on connecting more tightly distinct oxidant species and their enzymatic sources to physiological or pathophysiological processes. Another important factor will be uncovering and manipulating specific microenvironments. The chemical milieu, in particular oxygen availability, is a limiting factor for ROS production, but the hypoxia developing due to ROS production (e.g., *via* the neutrophil oxidative burst) in a low oxygen environment can have beneficial effects such as resolution of inflammation and accelerated tissue restitution (133). The presence and concentration of H_2_O_2_ and secondary oxidants (hypochlorous acid, peroxynitrite) will modify the barrier environment and the interactions between host and microbiota, leading to changes in microbiota diversity, composition, and the microbial metabolome that can be beneficial or harmful for the host. Case in point is the intestinal dysbiosis and appearance of pathobionts in NADPH oxidase deficient mice (94, 113). The nanomolar H_2_O_2_ released from mucosal barriers is inadequate for bactericidal activity, but its interference with the fitness and virulence of certain microorganisms supports its use as a promising alternative to antibiotics (273, 274). However, this antivirulence strategy will require the identification and targeting of specific pathogens to avoid the subversion of these oxidants for their own advantage as reported for some pathogenic bacteria (275–281).

Given the essential role of ROS for basic physiological functions, future therapeutics should include careful modulation of the redox state. Therapeutic interest today focuses on the margins of the ROS continuum, namely anti- or pro-oxidant interventions, but new approaches to modify physiological redox processes will be vital for improving health and well-being. Current limitations are the lack of targeted drugs modulating the activity of specific enzyme isoforms or of drugs that can adapt their mode of action by sensing ROS levels in situ. Sophisticated delivery systems for novel small molecule- or peptide-based activators will help restoring or enhancing beneficial ROS (mainly H_2_O_2_) at a predetermined location in order to achieve a positive outcome and to prevent undesired side effects linked to toxicity. For this purpose, exploitation of natural properties of commensals or their genetic modification, discovery of agonists stimulating ROS-generating enzymes, design of functional materials and delivery vehicles releasing H_2_O_2_, for example by utilizing recombinant enzyme-substrate pairs, will provide therapeutic options for infections, inflammatory diseases, and regenerative medicine. Next generation treatments will likely include strategies for altering the chemical environment at defined locations in a sustainable manner, with ROS being one of the key targets.

## Author Contributions

Concept (UK). Writing and editing (AD, UK). Figure design (AD). Funding (UK). All authors contributed to the article and approved the submitted version.

## Conflict of Interest

The authors declare that the research was conducted in the absence of any commercial or financial relationships that could be construed as a potential conflict of interest.
